# Supplementary material for: Continuum of care for maternal health in Uganda: A national cross-sectional study
Source: PLoS One. 2022 Feb 24;17(2):e0264190. doi: 10.1371/journal.pone.0264190 (PMC8870527; doi:10.1371/journal.pone.0264190)
Supplement: S1 Table — (DOCX) [file pone.0264190.s001.docx]

**Supplementary table 1: Trends in continuum of care utilization in Uganda over the last two decades**

| **Variable** | **2001 N=4,489 (%)** | **2006 N=5,035(%)** | **2011 N=4,968 (%)** | **2016**  **N= 10,152 (%)** |
| --- | --- | --- | --- | --- |
| **ANC frequency** | | | | |
| Less than 4 | 2602 (58.0) | 2601 (52.8) | 2601 (52.4) | 4072 (40.1) |
| 4 and above | 1881 (42.0, 95% CI: 39.9-42.9) | 2377 (47.2, 95% CI: 46.1- 48.8) | 2366 (47.6, 95% CI: 45.9- 48.8) | 6080 (59.9,95% CI: 59.0-60.8) |
| **Place of delivery^b^** | | | | |
| Home | 2712 (60.7) | 2743 (54.5) | 1931 (38.9) | 2372 (23.4) |
| Health facility | 1756 (39.3, 95% CI: 37.2-40.2) | 2291 (45.5, 95% CI: 44.4-47.0) | 3034 (61.1, 95% CI: 60.1-62.9) | 7780 (76.6,95% CI:75.8-77.5) |
| **PNC^c^** | | | | |
| No | 2526 (92.3) | 2226 (80.2) | 3331 (68.6) | 7872 (77.5) |
| Yes | 212 (7.7, 95% CI: 6.7-8.9) | 549 (19.8, 95% CI: 18.0-20.9) | 1529 (31.4, 95% CI: 30.2-32.9) | 2280 (22.5, 95% CI: 21.5-23.2) |
| **Continuum of Care** | | | | |
| No | 4,489 (100) | 5033 (100) | 4382 (88.2) | 9061 (89.3) |
| Yes | 0 (0, 95% CI: 0.0-0.1) | 2 (0, 95% CI: 0.0-0.1) | 586 (11.8, 95% CI: 11.0-12.8) | 1091 (10.7, 95% CI: 10.0-11.2) |

^a^ Missing 6 IN 2001, 2 in 2006, 1 in 2011, ^b^Missing 21 IN 2001, 1 in 2006, 3 in 2011 and ^c^Missing 1751 in 2001, 2260 in 2006, 107 in 2011
