# Supplementary material for: Continuum of care for maternal health in Uganda: A national cross-sectional study
Source: PLoS One. 2022 Feb 24;17(2):e0264190. doi: 10.1371/journal.pone.0264190 (PMC8870527; doi:10.1371/journal.pone.0264190)
Supplement: S2 Table — (DOCX) [file pone.0264190.s002.docx]

**Table 3: Predictors of complete continuum of maternity care in Uganda**

| **Category** | **Incomplete COC**  **n=9061** | **Complete COC**  **n=1091** | **Crude model**  **COR (95%CI)** | **P-value** | **Adjusted model II**  **AOR (95%CI)**  **N=8044** |
| --- | --- | --- | --- | --- | --- |
| **Age** |  |  |  | 0.002 |  |
| 35 to 49 | 2004 (22.1) | 178 (16.3) | 1 |  | 1 |
| 25 to 34 | 3897 (43.0) | 528 (48.4) | **1.53 (1.20-1.95)** |  | 1.17 (0.89-1.55) |
| 15 to 24 | 3160 (34.9) | 385 (35.3) | **1.38 (1.11-1.71)** |  | 0.97 (0.69-1.39) |
| **Residence** |  |  |  | **<0.001** |  |
| Urban | 1968 (21.7) | 378 (34.6) | 1 |  | 1 |
| Rural | 7093 (78.3) | 713 (65.4) | **0.52 (0.42-0.66)** |  | 0.94 (0.71-1.25) |
| **Region** |  |  |  | <0.001 |  |
| East | 2485 (27.4) | 242 (22.2) | 1 |  | 1 |
| North | 1846 (20.4) | 215 (19.7) | 1.20 (0.95-1.50) |  | 1.24 (0.96-1.60) |
| West | 2414 (26.6) | 145 (13.3) | 0.62 (0.46-0.83) |  | **0.58 (0.43-0.79)** |
| Central | 2316 (25.6) | 490 (44.9) | **2.17 (1.68-2.82)** |  | **1.47 (1.09-1.96)** |
| **Parity** |  |  |  | <0.001 |  |
| 5 and above | 3190 (35.2) | 263 (24.1) | 1 |  | 1 |
| 2-4 | 4111 (45.4) | 539 (49.4) | **1.59 (1.32-1.92)** |  | 1.12 (0.88-1.43) |
| 1 | 1760 (19.4) | 289 (26.5) | **1.99 (1.58-2.50)** |  | 1.32 (0.94-1.85) |
| **Household Size** |  |  |  | <0.001 |  |
| 6 and Above | 4588 (50.6) | 474 (43.4) | 1 |  | 1 |
| Less than 6 | 4473 (49.4) | 617 (56.6) | **1.34 (1.16-1.55)** |  | 1.08 (0.89-1.31) |
| **Working status** |  |  |  | 0.615 |  |
| Working | 7145 (78.9) | 871 (79.8) | 1 |  | - |
| Not working | 1916 (21.1) | 220 (20.2) | 0.94 (0.76-1.18) |  | - |
| **Marital status** |  |  |  | 0.680 |  |
| Not Married | 1698 (18.7) | 198 (18.1) | 1 |  | - |
| Married | 7363 (81.3) | 894 (81.9) | 1.04 (0.86-1.27) |  | - |
| **Education Level** |  |  |  | <0.001 |  |
| No education | 994 (11.0) | 68 (6.2) | 1 |  | 1 |
| Primary Education | 5606 (61.9) | 484 (44.4) | **1.26 (0.96-1.65)** |  | 1.10 (0.81-1.49) |
| Secondary Education | 1934 (21.3) | 351 (32.1) | **2.64 (1.99-3.51)** |  | **1.50 (1.03-2.17)** |
| Tertiary | 527 (5.8) | 188 (17.2) | **5.19 (3.69-7.29)** |  | 1.64 (0.95-2.82) |
| **Wealth Index** |  |  |  | <0.001 |  |
| Richest | 1753 (19.3) | 425 (39.0) | 1 |  | 1 |
| Richer | 1658 (18.3) | 204 (18.7) | **0.51 (0.39-0.66)** |  | 1.01 (0.71-1.45) |
| Middle | 1785 (19.7) | 136 (12.5) | **0.31 (0.24-0.41)** |  | 0.95 (0.64-1.39) |
| Poorer | 1921 (21.2) | 153 (14.0) | **0.33 (0.26-0.42)** |  | 1.03 (0.70-1.52) |
| Poorest | 1944 (21.5) | 173 (15.9) | **0.37 (0.29-0.47)** |  | 1.11 (0.73-1.70) |
| **Exposure to Radio** |  |  |  | **<0.001** |  |
| **Not at all** | **2458 (27.1)** | **209 (19.2)** | **1** |  | **1** |
| **Less than once a week** | **1384 (15.3)** | **167 (15.3)** | **1.42 (1.10-1.84)** |  | **1.27 (0.94-1.72)** |
| **At least once a week** | **5219 (57.6)** | **715 (65.5)** | **1.61 (1.34-1.94)** |  | **1.11 (0.88-1.41)** |
| **Exposure to Newspapers** |  |  |  | <0.001 |  |
| Not at all | 7501 (82.8) | 686 (62.9) | 1 |  | 1 |
| Less than once a week | 1008 (11.1) | 201 (18.4) | **2.18 (1.73-2.75)** |  | 1.24 (0.94-1.64) |
| At least once a week | 552 (6.1) | 204 (18.7) | **4.04 (3.32-4.92)** |  | 1**.71 (1.24-2.34)** |
| **Exposure to TELEVISION** |  |  |  | <0.001 |  |
| Not at all | 6628 (73.1) | 582 (53.3) | 1 |  | 1 |
| Less than once a week | 965 (10.7) | 140 (12.8) | **1.65 (1.31-2.08)** |  | 1.09 (0.80-1.48) |
| At least once a week | 1468 (16.2) | 369 (33.8) | **2.86 (2.31-3.56)** |  | **1.40 (1.01-1.95)** |
| **Husband’s education level^a^** |  |  |  | <0.001 |  |
| None | 474 (6.6) | 43 (5.0) | 1 |  | 1 |
| Primary | 4015 (55.9) | 331 (38.4) | 0.92 (0.65-1.31) |  | 0.85 (0.59-1.22) |
| Secondary | 1922 (26.8) | 283 (32.8) | **1.64 (1.13-2.39)** |  | 1.04 (0.69-1.56) |
| Tertiary | 769 (10.7) | 206 (23.9) | **2.98 (2.03-4.36)** |  | 1.25 (0.80-1.94) |
| **Sex of household head** |  |  |  | 0.444 |  |
| Male | 6642 (73.3) | 783 (71.8) | 1 |  | - |
| Female | 2419 (26.7) | 308 (28.2) | 1.08 (0.89-1.31) |  | - |
| **Timing of ANC** |  |  |  | <0.001 |  |
| Above 3 months | 6517 (71.9) | 396 (36.4) | 1 |  | 1 |
| 1- 3 months | 2544 (28.1) | 695 (63.6) | **1.46 (1.22-1.76)** |  | **1.42 (1.17-1.73)** |
| **Age at first birth** |  |  |  | 0.001 |  |
| 18 and above | 5635 (62.2) | 747 (68.5) | 1 |  | 1 |
| Less than 18 | 3426 (37.8) | 344 (31.5) | **0.76 (0.64-0.90)** |  | 1.08 (0.88-1.32) |
| **Religion** |  |  |  | 0.118 |  |
| Catholics | 3576 (39.5) | 427 (39.1) | 1 |  | 1 |
| Others | 270 (3.0) | 32 (2.9) | 0.98 (0.64-1.49) |  | 1.20 (0.76-1.89) |
| Anglican | 2832 (31.3) | 322 (29.5) | 0.95 (0.80-1.14) |  | 0.94 (0.76-1.15) |
| Pentecostal | 1163 (12.8) | 122 (11.2) | 0.88 (0.68-1.14) |  | 0.73 (0.54-0.99) |
| Muslims | 1220 (13.5) | 189 (17.3) | **1.30 (1.03-1.64)** |  | 0.94 (0.71-1.23) |
| **Care seeking decision** |  |  |  | 0.476 |  |
| Woman alone | 2099 (28.5) | 273 (30.6) | 1 |  | - |
| Woman and Someone | 3234 (43.9) | 389 (43.6) | 0.93 (0.77-1.11) |  | - |
| Not involved | 2030 (27.6) | 231 (25.9) | 0.88 (0.70-1.10) |  | - |
| **Contraception use** |  |  |  | <0.001 |  |
| No | 5569 (61.5) | 578 (53.0) | 1 |  | 1 |
| Yes | 3492 (38.5) | 513 (47.0) | **1.42 (1.21-1.66)** |  | 1.18 (0.99-1.40) |

**Bold** significant at p-value less than 0.05
