# Supplementary material for: Continuum of care for maternal health in Uganda: A national cross-sectional study
Source: PLoS One. 2022 Feb 24;17(2):e0264190. doi: 10.1371/journal.pone.0264190 (PMC8870527; doi:10.1371/journal.pone.0264190)
Supplement: Attachment — Submitted filename: Review_Continuum of maternity care for maternal and newborn health in Uganda- a national cross-sectional study.docx [file pone.0264190.s003.docx]

**Continuum of maternity care for maternal and newborn health in Uganda: a national cross-sectional study**.

**Overall comment**

Overall, the authors discussed the important topic of the continuum of care for maternal and newborn health in Uganda. They conducted a secondary analysis of the 2016 Demographic and Health Survey to determine the prevalence and factors associated with the continuum of maternity care in Uganda. There was a general lack of interpretation of results regarding the discontinuities between antenatal care, health facility childbirth, and postnatal care services. The associated factors, such as initiation of ANC in the first trimester, level of education, and exposure to mass media, have been reported in several studies. Therefore, it would be great if the authors could add evidence and implication for policy makers based on findings of this study.

Please find my comments in blue.

**Abstract**

- Results: About 59.9% (6080/7262) (95% CI: 59.0-60.8) had four or more antenatal visits. -Please check if the denominator is correct. I think you do not need to report exact numbers every time if you report results with a percentage with the denominator.

**Introduction**

- The first paragraph seems too long. It needs to be restructured to provide an overview of maternal, neonatal and under-5 childhood deaths by comparing sub-Saharan Africa with the other regions of the world.
- Almost all 277,300 (94%) of the annual maternal deaths occur in low and middle-income countries and roughly two-thirds (196,000) of these deaths happen in South East Asia and sub- Saharan Africa [2,3]. *-* How many annual maternal deaths occurred? When you report percentages, please indicate their denominators together.
- It is estimated that 45% of all the under-5 childhood deaths happen early in the neonatal period, especially in the first seven (7) days of life*.* - Is it the global situation or and sub-Saharan Africa?
- In introduction, you need to describe the situations of Uganda in terms of maternal, neonatal and under-5 childhood deaths if available. It is also recommended to include country data related to the study objectives briefly, such as the number of women of reproductive age, ANC and PNC attendance. It would provide rationales for the study objectives and the interventions for the continuum of maternity care recommended in the discussion.
- The current study focused on the completion of the continuum of care by specifically looking at antenatal care (ANC) attendance, health facility utilization during childbirth, and postnatal care (PNC). – This sentence in the second paragraph should be moved to the next paragraph as it describes the study objectives.
- It is better to include evidence available on the association between the continuum of maternity care and maternal and newborn health if available in the second paragraph.

**Materials and Methods**

- Study design: it should be mentioned that this study is the secondary analysis of the nationally-representative cross-sectional study (UDHS) conducted between June and December 2016.
- Sample size and sampling: Did all the sample of 10,152 women have the outcome data? If you excluded some women due to missing data on the outcome, please explain it or add a study population flow chart.
- Outcome Variable: Complete continuum of care was the outcome variable. Complete continuum of maternity care was considered when a woman reported having had: at least four ANC (ANC4+) contacts during the most recent pregnancy, utilized a health facility during the most recent childbirth and had at least one postnatal check-up within six weeks after childbirth: It would be great if you could add definitions of each segment consisting continuum of care. Are they in line with the Uganda MOH/WHO’s recommendations, for instance, in terms of the number and quality of ANC visits, supervision of a skilled service provider and length of stay in a health facility during childbirth and timing of postnatal check-up?
- Independent variables: Some variables included in the analyses and the result tables were not listed here, such as contraceptive use and religion. For variable selection, it is better to refer to a conceptual framework, such as Owilli’s continuum of care framework for maternal, newborn and child health (https://doi.org/10.1186/s12889-016-3075-0).

**Results**

- Characteristics of the study population: If you describe 95% CI in the narrative part, it is better to include them in Table 1 as well. Also, recheck Table 1 as total numbers exceed the sample size for some variables (i.e., postnatal care, residence).
- Of the 10,125 women, 59.9% (6,080/7262) (95% CI: 59.0-60.8) had four or more ANC contacts while 76.6% (7,780/10,125) (95% CI: 75.8-77.5) utilized health facility for childbirth and 22.5% (2,280/10,125) (95% CI: 21.5-23.2) attended postnatal care within six weeks after childbirth. – Is the denominator for four or more ANC visits correct?
- Factors associated with complete continuum of maternity care: The description is too short. You need to explain the key statistical outputs provided in Table 2. Which model is the main model that you used to discuss the results? In the description, were those independent variables associated with the outcome negatively or positively? For this, I think it is important to include odds ratios for some key independent variables.
- Throughout the manuscript, there were inconsistencies in reporting formats and typos, such as the use of thousand separators. Please review the whole manuscript.

**Discussion**

- We found a low level of utilization of complete continuum of maternity care at 11%. The level of use was highest for the place of childbirth at (7,789/10,125):77%), followed by ANC 4 or more visits at (6,080/7,262: 60%) and lowest for PNC at (2,280/1, 0125: 23%). - The discussion of the study seems mixed with results. This sentence was already presented in the results. You need to add more interpretation of the findings in the discussion.
- Uganda’s levels of completion of the continuum of maternity care is similar to that of other countries in the region. For both Ethiopia and Tanzania, the reported level is at about 10% [27,29], 8% for Ghana [28] and 7% for Lao [12]. Compared to Kenya, it is more than three times below the reported level of completion of the continuum of maternity care at 36% [32]. - You compared the prevalence of a complete continuum of maternity care among several countries. Did these studies use the same definition of the continuum of maternity as one used for this study? Moreover, are they all national-level studies? Please note the level of continuum of care may vary even within a country. Therefore, if you want to compare the prevalence of continued care with other studies, they should be nationally representative data. Otherwise, please specify locations, for instance, districts and regions.
- The largest dropout of 54% occurred between the stages of childbirth and the postnatal period. - I think it would be useful if you could provide more insights on this point by comparing it with the situations of other counties. For instance, how about the situation in Kenya?
- In the health sector, the decentralization policy has greatly improved physical access because it is estimated that 72% of the population lives within five kilometers of a health facility [34].This could partly explain the observed increase in the number health facility deliveries because the basic emergency obstetric care services have been brought closer to the community. - This paragraph is confusing. This study used only UDHS 2016 data, so you were not able to explore changes over time. If you want to discuss the increase in the number of health facility deliveries, you need to present the baseline. You did not compare longitudinal data and present any data about health facility delivery in the past in this manuscript. Decentralization policy may have increased all the ANC, health facility deliveries, PNC. This policy was not discussed before, so you need to explain this.
- Our results show an increase of 16% between the attendance of four or more ANC visits and health facility delivery… On the other hand, the observed drop in utilization between childbirth and postnatal care could be a pointer to the quality of service received during childbirth and labour. - They are directly related to the continued care. It would be useful if you could elaborate on why these discontinuities occurred, apart from the poor quality of maternity care in Uganda.
- Most of the factors associated with the continued care, such as initiation of ANC in the first trimester, level of education, and exposure to mass media, were reported and examined in previous studies. It is great if you could add evidence to them based on the findings of this study. At the same time, you described each associated factor using one paragraph, which is a bit wordy. It is great you can summarize discussions and put more focus on the unique findings of your study.
- You found large geographic variations in the continuum of maternity care. It is interesting if you can further investigate why the Eastern area, the poorest region with some of the poorest maternal and child health indicators, had a better continuum of care than the Western area. For instance, are there development partners supporting the continuum of care in this region?
- This finding may partly be explained by the unique cultural practices and beliefs about pregnancy and childbirth in this region – Please provide a few examples here.

**Discussion**

- I think it would be great if you could include recommendations beyond mass media and education programs to promote the complete continuum of care, such as support for early initiation of ANC and the integration of ANC and PNC programs based on your findings.
