# Supplementary material for: Continuum of care for maternal health in Uganda: A national cross-sectional study
Source: PLoS One. 2022 Feb 24;17(2):e0264190. doi: 10.1371/journal.pone.0264190 (PMC8870527; doi:10.1371/journal.pone.0264190)
Supplement: Attachment — Submitted filename: Respnse to reviewers comments.docx [file pone.0264190.s004.docx]

24^th^/11/2019

To

Dr. Masamine Jimba

Academic Editor

PLOS ONE

Dear Dr. Masamine Jimba

Re; **Response to reviewers’ comments and resubmission of revised manuscript PONE-D-21-14873**

Thank you for taking off time to review and provide feedback on this manuscript titled **“Continuum of maternity care for maternal and newborn health in Uganda: a national cross-sectional study”**. Please receive the revised manuscript and a point-by-point response to the comments raised by the peer reviewers as summarized in the table below.

| **Comment** | **Response** | **Line** |
| --- | --- | --- |
| **Reviewer #1** | | |
| Thank you for giving me a chance to review your manuscript. The manuscript shows the latest status of continuum of care including antenatal care visit, place of child birth, and postnatal care visit in Uganda very clearly. However, I have two major and several minor suggestions as follow. I hope my comments will help to improve the manuscript. | We are grateful for the time you dedicated to provide feedback to improve this manuscript. | NA |
| **Major suggestions** | | |
| 1. I understand that many previous studies measured the complete continuum of care with the same definition used in this study, and DHS collects data on four antenatal care visits and postnatal check within 6-week postpartum. However, WHO recommends at least eight times of antenatal care visits, and four times of postnatal care visits. If the national guidelines in Uganda apply the WHO’s recommendation, this study should measure continuum of care based on the guidelines used at the time of survey. | Thank you so much for the observation. This survey was conducted between 2011 and 2016 (five years preceding the survey) yet the WHO guidelines of at least 8 times was introduced globally in 2016. Relatedly, the MoH in Uganda has just changed its ANC guidelines in the last two years towards the eight ANC contacts. So, we decided to use the guidelines that were being implemented at the time of the survey. [Goal Oriented Antenatal Care Protocol \| Ministry of Health Knowledge Management Portal](http://library.health.go.ug/publications/sexual-and-reproductive-health/goal-oriented-antenatal-care-protocol). This information has been provided under the sections on study setting and independent variables. | 119 and 159 |
| 2. As reading the second paragraph of the discussion section, I thought that readers would like to see the change in antenatal care visits, place of childbirth, postnatal care visit, and continuum of care over years. Because Uganda DHS 2000-01, 2006, and 2011 DHS are available, I would like to encourage authors to add these results, which will show the effect of centralization policy and socio-economic development on utilization of maternity care services in the country. | Thank you for the observation. This is something that we had done in the analysis but decided not to include in this manuscript because there were a lot of inconsistencies in the data. However, we have now included this information in a supplementary table 1 to show the trends. Although, we think this does not show the effect of centralization policy and socio-economic development on utilisation of maternity care services in the country as the reviewer suggests. | 227-228 |
| **Minor suggestions** | | |
| 1. Please correct “the latest Uganda Demographic and Health Survey” to “Uganda Demographic and Health Survey 2016”. | This has been corrected in the methods section of abstract. | 21 |
| 2. Please report AOR and 95% with the following way: (AOR, 95%CI) | This has been corrected | 29 -37  164 -174 |
| 3. In the first paragraph. I wonder the references #10. #11, #12, and #13 provide any evidence that complete continuum of care prevented or reduced maternal and newborn deaths. | Thank you for this query, these papers review the literature on the rational for and importance of the continuum of care in reducing maternal and perinatal morbidity and mortality. Although, they all aim to identify factors associated with completion of the continuum of maternity care | 56 - 60 |
| 4. In the second paragraph, please add the information on how many times and when mothers and their newborns should receive postnatal checkups in the national guidelines in Uganda. | The following statement has been added “For home births, the first postnatal check should occur within 24 hours of birth. Thereafter, all mothers and newborns should receive at least three additional postnatal checks on day three, between day seven and 14, and between week two and six following child birth” | 72-74 |
| 5. In the third paragraph, please show the factors associated with each of the maternal child health care services in Uganda that were reported by previous studies. | These factors have been added to the third paragraph as suggested. | 84 - 94 |
| Materials and Methods | | |
| 6. In the study setting section, please provide more detail about the situation in Uganda, including maternal and child health indicators, health system, or the characteristic of each region. | Thank you for the suggestion, these details have been added. | 110 - 134 |
| 7. Please correct the sample size of 10,125 to 10,152. | Sorry for the typo, this has now been corrected. | 144 |
| 8. Please report the frequency of antenatal care visits, followed by place of childbirth and postnatal care. | We reported these in this order both in the abstract and results. | 30-31  191-192 |
| 9. On Table 2, please report the percentage of complete CoC for each category by row. For example, the percentage of complete CoC among women aged 15-24 by row is 10.9% (385/3546). | Thank you for the suggestion. However, we present the preferred column percentages in all our tables | 216 - 217 |
| 10. Please provide the sample size for the adjusted model I and II, respectively. | Added in table 2 | 216 - 217 |
| 11. In the first paragraph, please report percentage only, and delete the values of numerator and denominator indicated after the percentage. | This has been done as advised | 225-226 |
| 12. In the fourth paragraph, authors mentioned that Western Uganda has unique cultural practice and belief about pregnancy and childbirth. Please explain more details. | For instance, anecdotal evidence from this region suggests that for the first seven days after childbirth, the baby should not be seen by strangers. So, babies born at home are kept indoors until the first week of life has elapsed. While those that give birth normally in health facility are in a rush to get back home early to full fill this cultural practice because the health facility does not offer adequate privacy. | 301-306 |
| **Reviewer #2** | | |
| Overall, the authors discussed the important topic of the continuum of care for maternal and newborn health in Uganda. They conducted a secondary analysis of the 2016 Demographic and Health Survey to determine the prevalence and factors associated with the continuum of maternity care in Uganda. There was a general lack of interpretation of results regarding the discontinuities between antenatal care, health facility childbirth, and postnatal care services. The associated factors, such as initiation of ANC in the first trimester, level of education, and exposure to mass media, have been reported in several studies. Therefore, it would be great if the authors could add evidence and implication for policy makers based on findings of this study.  Please find my comments in blue in the attachment. | Thank you for taking time to review this manuscript and provide comprehensive feedback to make it better. We believe that we have addressed all the issues raised by the reviewers. | NA |
| **Abstract** | | |
| Results: About 59.9% (6080/7262) (95% CI: 59.0-60.8) had four or more antenatal visits. -Please check if the denominator is correct. I think you do not need to report exact numbers every time if you report results with a percentage with the denominator. | Thank you for the observation. This was an error that we have corrected. The denominator should be 10,152. Your suggestion has been adopted. | 29-32 |
| **Introduction** | | |
| The first paragraph seems too long. It needs to be restructured to provide an overview of maternal, neonatal and under-5 childhood deaths by comparing sub-Saharan Africa with the other regions of the world. | We have modified the paragraph accordingly | 225-254 |
| Almost all 277,300 (94%) of the annual maternal deaths occur in low and middle-income countries and roughly two-thirds (196,000) of these deaths happen in South East Asia and sub- Saharan Africa [2,3]. *-* How many annual maternal deaths occurred? When you report percentages, please indicate their denominators together. | Thank you for the advice, this has been implemented | 45 - 46 |
| It is estimated that 45% of all the under-5 childhood deaths happen early in the neonatal period, especially in the first seven (7) days of life*.* - Is it the global situation or and sub-Saharan Africa? | This is in low- and middle-income countries. | 55 |
| In introduction, you need to describe the situations of Uganda in terms of maternal, neonatal and under-5 childhood deaths if available. It is also recommended to include country data related to the study objectives briefly, such as the number of women of reproductive age, ANC and PNC attendance. It would provide rationales for the study objectives and the interventions for the continuum of maternity care recommended in the discussion. | Thank you for this suggestion. We have provided this information under the study setting section. | 110 - 134 |
| The current study focused on the completion of the continuum of care by specifically looking at antenatal care (ANC) attendance, health facility utilization during childbirth, and postnatal care (PNC). – This sentence in the second paragraph should be moved to the next paragraph as it describes the study objectives. | This suggestion is noted and it has been implemented accordingly | 98-100 |
| It is better to include evidence available on the association between the continuum of maternity care and maternal and newborn health if available in the second paragraph. | This evidence has been included here “It is estimated that 80% of maternal deaths and 66% of neonatal deaths in the world could be averted by ensuring that women have access to quality and effective maternity care services especially during childbirth and in the first week of life [9,10].” | 56-58 |
| Materials and Methods | | |
| Study design: it should be mentioned that this study is the secondary analysis of the nationally representative cross-sectional study (UDHS) conducted between June and December 2016. | This has been added. | 141 |
| Sample size and sampling: Did all the sample of 10,152 women have the outcome data? If you excluded some women due to missing data on the outcome, please explain it or add a study population flow chart. | No missing data on the outcome was noted. However, PNC had 44 participants, ANC 56 who said, “I don’t know” so these were considered as if they had no PNC check and had less than 4 visits, respectively. Place of delivery had no I do not know responses. | 143-145 |
| Outcome Variable: Complete continuum of care was the outcome variable. Complete continuum of maternity care was considered when a woman reported having had: at least four ANC (ANC4+) contacts during the most recent pregnancy, utilized a health facility during the most recent childbirth and had at least one postnatal check-up within six weeks after childbirth: It would be great if you could add definitions of each segment consisting continuum of care. Are they in line with the Uganda MOH/WHO’s recommendations, for instance, in terms of the number and quality of ANC visits, supervision of a skilled service provider and length of stay in a health facility during childbirth and timing of postnatal check-up? | 1. Until about two years ago, ANC guidelines in Uganda recommended at least 4 ANC visits for every pregnant woman.  2. Each pregnant woman is expected/recommended to give birth under the care of skilled birth attendant  3. The same guidelines recommend that each woman should have at least four checks in the postnatal period. However, one of the limitations of the UDHS data is that it only asks about only one PNC visit. So, information about the other three visits is not available. | 151-157 |
| Independent variables: Some variables included in the analyses and the result tables were not listed here, such as contraceptive use and religion. For variable selection, it is better to refer to a conceptual framework, such as Owilli’s continuum of care framework for maternal, newborn and child health (https://doi.org/10.1186/s12889-016-3075-0). | Thank you for the observation and for pointing us to this conceptual frame work to refer to. Variables that were not listed in the methods have now been listed to include those in Owilli’s framework, which we had measured. | 172-173  187-188 |
| **Results** | | |
| Characteristics of the study population: If you describe 95% CI in the narrative part, it is better to include them in Table 1 as well. Also, recheck Table 1 as total numbers exceed the sample size for some variables (i.e., postnatal care, residence). | Thanks for the observation. This has been corrected in table 1 | 204-205 |
| Of the 10,125 women, 59.9% (6,080/7262) (95% CI: 59.0-60.8) had four or more ANC contacts while 76.6% (7,780/10,125) (95% CI: 75.8-77.5) utilized health facility for childbirth and 22.5% (2,280/10,125) (95% CI: 21.5-23.2) attended postnatal care within six weeks after childbirth. – Is the denominator for four or more ANC visits correct? | Thanks this has been corrected. | 195-199 |
| Factors associated with complete continuum of maternity care: The description is too short. You need to explain the key statistical outputs provided in Table 2. Which model is the main model that you used to discuss the results? In the description, were those independent variables associated with the outcome negatively or positively? For this, I think it is important to include odds ratios for some key independent variables. | Thank you for the important observation, these have now been provided as advised. | 207-217 |
| Throughout the manuscript, there were inconsistencies in reporting formats and typos, such as the use of thousand separators. Please review the whole manuscript. | The whole manuscript has been revised to standardize the writing. | NA |
| **Discussion** | | |
| We found a low level of utilization of complete continuum of maternity care at 11%. The level of use was highest for the place of childbirth at (7,789/10,125):77%), followed by ANC 4 or more visits at (6,080/7,262: 60%) and lowest for PNC at (2,280/1, 0125: 23%). - The discussion of the study seems mixed with results. This sentence was already presented in the results. You need to add more interpretation of the findings in the discussion. | Thank you for this advice, it has been implemented | 229-231 |
| Uganda’s levels of completion of the continuum of maternity care is similar to that of other countries in the region. For both Ethiopia and Tanzania, the reported level is at about 10% [27,29], 8% for Ghana [28] and 7% for Lao [12]. Compared to Kenya, it is more than three times below the reported level of completion of the continuum of maternity care at 36% [32]. - You compared the prevalence of a complete continuum of maternity care among several countries. Did these studies use the same definition of the continuum of maternity as one used for this study? Moreover, are they all national-level studies? Please note the level of continuum of care may vary even within a country. Therefore, if you want to compare the prevalence of continued care with other studies, they should be nationally representative data. Otherwise, please specify locations, for instance, districts and regions. | Thank you so much for this important observation. Corrections have been made to reflect these very specific details in the comparisons that have been made. | 232-238 |
| The largest dropout of 54% occurred between the stages of childbirth and the postnatal period. - I think it would be useful if you could provide more insights on this point by comparing it with the situations of other counties. For instance, how about the situation in Kenya? | This has been done | 239-246 |
| In the health sector, the decentralization policy has greatly improved physical access because it is estimated that 72% of the population lives within five kilometers of a health facility [34].This could partly explain the observed increase in the number health facility deliveries because the basic emergency obstetric care services have been brought closer to the community. - This paragraph is confusing. This study used only UDHS 2016 data, so you were not able to explore changes over time. If you want to discuss the increase in the number of health facility deliveries, you need to present the baseline. You did not compare longitudinal data and present any data about health facility delivery in the past in this manuscript. Decentralization policy may have increased all the ANC, health facility deliveries, PNC. This policy was not discussed before, so you need to explain this. | This is noted, in the study setting section, we have provided more details on the decentralized nature of health care services in Uganda. You rightly point out that decentralization should ideally increase utilisation of all services (ANC, facility births, PNC). In this regard, we have added a supplementary file to show the changes in maternal child health services over time. | 115 -139 |
| Our results show an increase of 16% between the attendance of four or more ANC visits and health facility delivery… On the other hand, the observed drop in utilization between childbirth and postnatal care could be a pointer to the quality of service received during childbirth and labour. - They are directly related to the continued care. It would be useful if you could elaborate on why these discontinuities occurred, apart from the poor quality of maternity care in Uganda. | This has been improved, in the discussion section | 250-261 |
| Most of the factors associated with the continued care, such as initiation of ANC in the first trimester, level of education, and exposure to mass media, were reported and examined in previous studies**.** It is great if you could add evidence to them based on the findings of this study. At the same time, you described each associated factor using one paragraph, which is a bit wordy. It is great you can summarize discussions and put more focus on the unique findings of your study. | Thank you for this observation, the key finding in this study is the low level of the continuum of care because of the sharp drop between the stages of child birth and utilisation of PNC services. The first two paragraphs in the discussion explore this finding. | 272-277 |
| You found large geographic variations in the continuum of maternity care. It is interesting if you can further investigate why the Eastern area, the poorest region with some of the poorest maternal and child health indicators, had a better continuum of care than the Western area. For instance, are there development partners supporting the continuum of care in this region? | Thank you for the suggestion, this has been added. | 311-312 |
| This finding may partly be explained by the unique cultural practices and beliefs about pregnancy and childbirth in this region – Please provide a few examples here. | These have been added | 305 - 310 |
| I think it would be great if you could include recommendations beyond mass media and education programs to promote the complete continuum of care, such as support for early initiation of ANC and the integration of ANC and PNC programs based on your findings. | Thank you for the advice, this has been added. | 333-334 |
| **Reviewer #3** | | |
| The authors analyzed the level of continuum of care in maternal, newborn, and child health in Uganda using a nationally representative data. The manuscript is easy to read, and the analysis is conducted with a widely-accepted method. The reviewer hopes that the following comments help the authors to improve the manuscript. | Thank you very much for these comments | NA |
| **Major comment** | | |
| 1. When did the Government of Uganda started recommending ANC eight times? The authors explained that “In conformity with the current World Health Organisation (WHO) guidelines, the Ministry of Health in Uganda recommends that all women attend at least eight ANC visits and have childbirth in a health facility supervised by a skilled service provider [16].” (in Page 2). However, since the data were collected in 2016, it is important to mention the guidelines in Uganda when the survey was conducted. In addition, this statement is not consistent with ANC component (ANC 4+) under the definition of continuum of care completion. | Thanks so much for the observation. This day was collected on births that occurred between 2011 and 2016 (five years preceding the survey) yet the WHO guidelines of at least 8 times was introduced globally in 2016. Hence, we decided to use the guidelines at the time when the outcome was occurring. | 72-75 |
| 2. What are the recommended timings of PNC in Uganda? Since it is related to how the continuum of care should be defined, tt is worth mentioned in the Introduction section. | This has been included as recommended. | 72-75 |
| 3. Why did the author define the PNC component of the continuum of care completion as “at least one postnatal check-up within six weeks after childbirth?” A major limitation of a continuum of care analysis using DHS dataset is lack of detailed variables on PNC. If this definition is not consistent with the PNC guidelines in Uganda, the authors may want to add this inconsistency as a limitation. | Thank you for the suggestion, we have added this in the limitation section. | 14/315-316 |
| 4. Is the variable for PNC based on PNC for mothers or PNC for children? Typically, DHS dataset has both of the variables. The authors need to explain the definition of the PNC component of the continuum of care completion clearly. | We used the women's dataset, in which the unit of analysis is the woman so we consider it as woman whose child had a postnatal check | 160-161 |
| 5. The authors mentioned that “The UDHS interviewed a total of 18,506 women aged 15–49 years” and “Our secondary analysis was a total enumeration of all the 10,152 women who had had a live birth within five years preceding the survey [23].” (in Page 3). And the authors analyzed 10,125 women according to the description in the Results section. The authors need to clarify why the number of women was limited to 10,125 from 18,506 or 10,152, either in the Methods or Results section. | Thank you for the observation. It was an error on our side to write 10,125 instead of 10, 152. We analyzed data of all 10,152 women who had a live birth 5 years preceding the survey. | 149 |
| 6. Eligible women included in the analysis seem to be different by indicators: complete continuum-of-care (10,125 women), four ANC or more (7,262), facility delivery (10,125), and (10,125 women). The column heading of Table 1 stated that N=10,152 and rows for place of delivery, postnatal care, anc visits, and complete continuum of care had 10,152 observations. Please check if these data are correctly analyzed and reported. | Thanks for the observation. It was an error on our side to write 10,125 instead of 10, 152. We analyzed data of 10,152 women who had a live birth 5 years preceding the survey. This has been corrected. | 149-150  221-222 |
| 7. How many women were included in the logistic regression analysis? The adjusted model II included the variables with missing observations, namely, husbands’ education level and care seeking decisions. Therefore, the number of women included in the adjusted models I and II may differ. Please clarify the number of women in the column heading of Table 2 (or any other appropriate place). | This has now been clarified in Table 2. | 221-222 |
| 8. Why did the Region variable have 4 categories only? According to DHS 2016 report (<https://dhsprogram.com/pubs/pdf/FR333/FR333.pdf>), there are 15 Regions. Although the continuum of care may be affected by supply-side characteristics, DHS dataset does not have such characteristics. A geographic area variable, such as the Region variable, can capture external factors that cannot be controlled by women’s and households’ characteristics in the regression model, including socioeconomic and healthcare service levels at the area level. It would be recommended not to merge the Region variable. Note that previous papers often use binary variables that capture geographic areas or multilevel models at the area level. | Thanks so much for the observation. The UDHS used sub-regions which were 15. However, in our analysis, we used region as a variable so we used the four regions of Uganda (Northern, Eastern, Central and Western) by combining the different sub-regions that make up regions.  Northern (Teso, Karamoja, Lango, Acholi, West Nile), Central (Kampala, Central 1 and Central 2), Eastern (Busoga, Bugishu and Bukedi) and Western (Tooro, Ankole, Bunyoro and Kigezi)  The same has also been used in other papers  (e.g Yang YY, Kaddu G, Ngendahimana D, Barkoukis H, Freedman D, Lubaale YA, Mupere E, Bakaki PM: Trends and determinants of stunting among under-5s: evidence from the 1995, 2001, 2006 and 2011 Uganda Demographic and Health Surveys. Public health nutrition 2018, 21(16):2915-2928). | 173 - 174 |
| **Minor comment** | | |
| 1. In the Abstract, please clarify the survey year (2016). | This has been done | 21 |
| 2. Introduction (Page 2): Data in the first paragraph (such as 277,300 maternal deaths, 1 in 59 160, or 5,400 pregnancies; 27 deaths per 1,000 livebirths, …) should be introduced with the year(s) of survey. | The suggestion has been taken and implemented accordingly | 45-51 |
| 3. In Table 2, the superscript “a” for the variable of “Husband’s education level” does not have a corresponding footnote. | Sorry for the error, this has been corrected | 221-222 |
| 4. In the first paragraph of the Discussion section, “54%” in the sentence, “The largest dropout of 54% occurred between the stages of childbirth and the postnatal period.” might have been 54 percentage points (76.6% - 22.5%). Note that 2.5%/76.6%=0.294 and it could be presented “71% dropout.” | Thank you for this brilliant observation, we have corrected this | 244 |
| 5. In the second paragraph of the Discussion section, “16%” in the sentence, “Our results show an increase of 16% between the attendance of four or more ANC visits and health facility delivery,” might have been 17 percentage points (76.6% - 59.9%). Note that 76.6%/59.9%=1.28 and it could be presented “28% difference.” | Thank you for this brilliant observation, we have corrected this. | 262 |
| **END** | | |
