# Supplementary material for: Continuum of care for maternal health in Uganda: A national cross-sectional study
Source: PLoS One. 2022 Feb 24;17(2):e0264190. doi: 10.1371/journal.pone.0264190 (PMC8870527; doi:10.1371/journal.pone.0264190)
Supplement: Attachment — Submitted filename: Respnse to reviewers comments -Revised.docx [file pone.0264190.s005.docx]

18^th^ /09/2021

To

Dr. Masamine Jimba

Academic Editor

PLOS ONE

Dear Dr. Masamine Jimba

Re; **Response to reviewers’ comments and resubmission of revised manuscript PONE-D-21-14873**

Thank you for taking off time to review and provide feedback on this manuscript titled **“Continuum of maternity care for maternal health in Uganda: a national cross-sectional study”**. Please receive the revised manuscript and a point-by-point response to the comments raised by the peer reviewers as summarized in the table below.

| **Comment** | **Response** | **Line** |
| --- | --- | --- |
| **Additional Editor Comments** | | |
| Thank you for your submission. Previous three reviewers asked you to revise many basic points and you revised most of them. This time, I would like to add my comments in addition to other reviewers’ further comments. | We are grateful for the time you dedicated to provide feedback to improve this manuscript. | NA |
| 1. L2: Your title includes “newborn health,” but it is not well taken up in the manuscript. Please consider to remove it. | The words “ and newborn” have been removed | 2,101,333 |
| 2. L18 and other lines: In many articles about the continuum of care in MNCH, “coverage” is used instead of “prevalence.” Please consider to use “coverage.” | Thank you for the suggestion, we have replaced the word prevalence with coverage in the whole manuscript. | 19,29,39,96 and 115 |
| 3. L23: In this manuscript, “continuum of care” and “continuum of maternal care” seem to be used interchangeably, which makes readers very confusing. Please be careful about using both phrases. | Sorry about this confusion, we have chosen to use “Continuum of maternity care” throughout the document. | Whole document |
| 4. L27: …“continuum of maternal care” or “continuum of care”? | We have chosen to use “Continuum of maternity care” throughout the document. | Whole document |
| 5. L32: ...attended “at least one” postnatal care… | Thank “at least one postnatal care visit” has been added | 34 |
| 6. L32: “A is associated with B” is not equal with “B is associated with A”. It might be better to write “The following factors were associated with the completion of the continuum of care” or …”…continuum of maternal care”: They were secondary level of education… | Thank you for the advice, this has been revised accordingly. | 35 - 40 |
| 7. L35: What do you mean by “print”? Newspapers and magazines? Or either one? | This has been clarified, it means both Newspapers and magazines | 39 |
| 8. L40: Out of many factors associated with the continuum of care or continuum of maternal care, only some factors were written in here. Are they because they were easily modifiable? Simple repetition of results are not recommendable in the conclusion section. | Yes, we chose the easily modifiable factors. This has now been stated more explicitly. | 42 - 47 |
| 9. L102: Spell out UDHS when it first appears. | This has been done | 106 |
| 10. L104: No need to use abbreviation such as UBOS, ICFPNFPs, PFPs, TCMPs as these abbreviations are not used after this. | Advice has been taken; all these abbreviations have been deleted | 133 - 134, 106 - 109 |
| 11. L119: Instead of “Generally,” you may write “In Uganda,” | Thank you, this has been done | 125 |
| 12. L136: …(UDHS)? Any way to rewrite it? | We have rewritten it to “This was a secondary data analysis of nationally representative cross-sectional survey data conducted between June and December 2016.” | 142 -143 |
| 13. L136 …secondary analysis or secondary data analysis? | We have revised it to secondary data analysis | 142 |
| 14. L139: In our secondary data analysis, we used data that were obtained from validated women’s… It is better to use “we” instead of using passive tense in the method section. Please recheck your whole method section from L139 to L189. | Thank you, we have revised this section accordingly | 148 - 206 |
| 15. L152: Again, “continuum of maternal care” or “continuum of care”? | We have chosen to use “Continuum of maternity care” throughout the document. | Whole document |
| 16. L163: …exposure to newspapers/magazines,… | We have revised this to media (Newspapers, media….. | 169 - 170 |
| 17. L179: …proportions…Use of capital letters is not appropriate. | Sorry about this, it has been corrected appropriately | 185 |
| 18. L196, 197, 198: 95% CI can be cut as it is available from the Table1. | These have been deleted | 201 - 205 |
| 19. L202: Table 1: Percent (%) and 95% CI? | Sorry for the omission, this has been corrected | 208 |
| 20. L207-L215: …“continuum of maternal care” or “continuum of care”? | We have chosen to use “Continuum of maternity care” throughout the document. | Whole document |
| 21. L226: …“continuum of maternal care” or “continuum of care”? | We have chosen to use “Continuum of maternity care” throughout the document. | Whole document |
| 22. L227: In this context, 10.7% is better. | 11% has been changed to 107% | 233 |
| 23. L225-237: The coverage of the continuum of care in different countries followed the same definition? Are they comparable? All of them were measuring “continuum of maternal care” or “continuum of care”? | Yes, they are comparable because they used the same definition for the continuum of maternity care. We crosschecked this in response to a comment by the reviewer from previous round of peer review comments. | 234 - 243 |
| 24. L232: 10.0% in Tanzania? | “level of coverage” has been added to make it complete. | 238 |
| 25. L233: 8.0% in Ghana? | “level of coverage” has been added to make it complete. | 240 |
| 26. L234: 7.0% in Lao PDR? | “level of coverage” has been added to make it complete. | 240 |
| 27. L234: Lao should be Lao PDR. | This change has been implemented | 240 |
| 28. L237: 36.0%? | 36.0% is the level of coverage in Western Kenya | 243-244 |
| 29. L250 to 255: This paragraph is well written. It is optional, but it might be better to write a recommendation not in each paragraph but at the end. The current one is somehow acceptable, though. | Thank you for the comment, we have chosen to leave it here since it is acceptable. | 259 - 264 |
| 30. L264-268: This sentence is redundant and it is better not to use a word ‘fact’ in this context. | This sentence “This low level of service utilisation could also be related to fact that many health facilities in Uganda do not have a clearly designated space for postnatal care as is the case with the ANC and childbirth.” Has been deleted. | 275 - 277 |
| 31. L275: A new phrase appears in here, “continued care.” Please use the same expression. | We have chosen to use “Continuum of maternity care” throughout the document. | 284 -285 |
| 32. L307-309: This topic sentence does not seem to make sense. Please discuss your data, instead of discussing ref 8, 38 and 40. | Thank you for advice, this has been revised accordingly | 320 - 322 |
| 33. L312: …“don’t” should be “do not”. | This has been changed | 321 |
| 34. L316: …radio or TV? Or both? In the results, it was TV only. | Thank you for the observation, it should be radio …… | 325 |
| 35. L312: Are you discussing maternal and newborn health or only maternal health? | As earlier suggested, we have adjusted it to maternal health | 324 |
| 36. L329: What do you mean by print? Newspapers and magazines? Or either one? | This has been clarified, it means both Newspapers and magazines | 338 |
| 37. L329: …television or TV? | We have replaced TV with television | 170,213 & 221 |
| 38. L329: …“continuum of maternal care” or “continuum of care” or “continuum of care for maternal and newborn health”? | We have chosen to use “Continuum of maternity care” throughout the document. | 341 |
| 39. L331: secondary analysis or secondary data analysis? The nuance is different. | We have revised it to analysis of secondary data | 340 |
| 40. L335: You mean study limitations? This should be a separate subheading like “Conclusion.” | Methodological considerations has been replace with “Study strengths and limitations” | 344 |
| 41. L347-8: … “continuum of maternal care” or “continuum of care” or “continuum of care for maternal and newborn health”? | We have chosen to use “Continuum of maternity care” throughout the document. | 357 - 358 |
| 42. L351: “being a resident of western region was positively…” This is misleading. | Thank you , the word “positively” has been removed | 360 |
| 43. L352: …”ministry of health” or “Ministry of Health”? | It should be “Ministry of Health” | 364 -365 |
| 44. L352: Low levels of what kind of education? Why only northern and eastern regions? | This has been revised to low level of formal education | 363, 368 -369 |
| 45. L355-356: This is not a complete English sentence. | We have now revised this sentence. | 363 - 366 |
| 46. L356-357: Is this relevant as a conclusion of this article? | This has been revised accordingly | 367 - 369 |
| 47. Reference section should be checked one by one very carefully. For example, the journal name of the reference 3 is wrong. When you use PDF files as references, please add access dates. Please do not rely on computer soft wares for making the reference section. | Advice taken and it has been implemented | 383 -565 |
| **Reviewer #1:** | | |
| Thank you for responding to my suggestions, and revising the manuscript. I saw the manuscript has been improved. However, I would to like to offer further suggestions to the authors | Thank for the positive comments and advise | NA |
| The descriptive result of place of childbirth, postnatal care, ANC visits, and complete continuum of care are the main finding of the study. I suggest the authors to make another table of these results to highlight them. Additionally, please show the results in the table with the following order: ANC visits, place of childbirth, postnatal care, and complete CoC. I found the data on ANC visits 4 and above is missed in Table 1. It is interesting to see the percentage of postnatal care among women delivered at facility and home, respectively, because the place of birth would affect the attendance of postnatal care. I saw that the authors preferred to presenting percentage by column in Table. I, which I accepted. If presenting the percentage by column, it will be better to show the percentage of each category for women who did not complete CoC too, because readers are interested in difference in the distributions between women who completed and did not complete CoC. | Thank you for the suggestions, it has been worked on and table 2 has been introduced to highlight the level of utilization of the different components of the COC.  The percentage of postnatal care among women delivered at facility and home, respectively has been added in the results section as advised.  The percentage of each category for women who did not complete CoC has also been added in Table 3. | 223 - 224  Lines 216 - 219  246-247 |
| Thank you for adding supplementary table 1. This is informative. Please briefly explain the calculations in the methods, results, and discussion sections. What do 56.3, 4.7, 44.2, and 10.9 mean in the cells of PNC 2001 and 2006. Why some results are reported with 95% CIs, and some are not. Please check missing values and clean the table. | We thank you for this advice, it has been adopted in the current manuscript. The supplementary table has been revised as follows; - We have added the missing 95% CI of the major outcome variables.  - The confusing 56.3, 4.7, 44.2 and 10.9 for postnatal care have been removed  As suggested the same information has been included in the methods results and discussion sections. | See supplementary table 1  See supplementary table 1  170 - 172 |
| Line23, Line105: Regarding sampling methods, the authors mentioned multistage stratified sampling in the abstract and stratified two-stage cluster sampling in the methods section. Please use one. | This has been corrected in the abstract | 24 |
| Line35, Line329: Please change print to newspaper. | This has been changed to Newspaper and magazine | 342 |
| Line81: Delete a double quotation. | The quotation has been removed | 93 |
| Line111: Please spell out 7.3M. | This has been done | 116 |
| Line115-116: Please use a reference to explain improved access to emergency obstetric and neonatal care services. | The reference has been provided | 122 |
| Line253: Please revise “every parturient should be …” to “every parturient and newborn should be…” | Thank you for the suggestion, this has been done | 262 |
| Line278: The effect of early initiation of ANC is widely known. I suggest the authors to discuss more about why women do not attend the first ANC in the first trimester, and potential interventions to improve the situation. | We done as suggested | 317 - 319 |
| **Reviewer #2:** | | |
| Thank you for addressing the reviewer’s comments. The revised paper is much improved, and the authors' responsiveness is much appreciated. I have only a few more comments: | Thank for the positive comments and advise | NA |
| 1. Please mention when the MoH Uganda started recommending at least eight ANC visit in the introduction (lines 64-67). Also, explain the guideline on ANC visits when the DHS data were collected, which recommended at least four ANC visit, in either study settings or outcome variables. | Thank you for the suggestions, these have been added appropriately as suggested. | 76  165 - 167 |
| 2. The current manuscript requires a detailed review of formatting. Please spell out abbreviations only when they are used for the first time (e.g., ANC, PNC) | This has been done throughout the document. | NA |
| 3. In “characteristics of the study population” in the results, I think you do not need to report exact numbers every time if you report percentages with their denominators, considering the word count. Please consider deleting exact numbers (i.e., 7,780/10,152). | Thank for the advice, the repeated denominators have been deleted | 199 - 207 |
| 4. The “Study setting” seems a bit too lengthy (lines 101-134). The authors are encouraged to revise the subsection focusing on key information related to the continuum of care for maternal and newborn health in Uganda. | Thank you for the suggestion, we revised it to remove some of the information. The extra description of the health care system in Uganda was requested for in the previous round of peer review. | 117- 121 |
| **Reviewer #3:** | | |
| The authors modified the manuscript by addressing errors and added explanations and interpretations. However, please note that the mark-up copy of the revised manuscript attached to the submission file was not fully consistent with the clean version, which made difficult for review the revisions. In the Response-to-reviewers file, the line numbers for some point-to-point responses may not be correct. | Sorry, about this confusion. We have revised this and hope that it is now resolved in the current document. | NA |
| 1. In the current submission, the authors presented the prevalence of individual components of services, namely ANC, place of delivery, and PNC, in Supplementary Table 1. Since the authors used these results extensively in the Discussion section, the authors seem to consider these results as important. The authors may want to describe the results of this supplementary table in the Results section. | Thank you for the suggestion, this has been added | 220 - 222 |
| 2. The authors seem to report the multivariable logistic regression analysis results of this study using Model II (N=8044) excluding those without currently married, not model I that included every eligible participant (N=10152). While the two models reported largely consistent results, the authors may want to use model I as the main result as it did not have loss of participants. They may use model II results as supplements (it simply shows that husbands’ education was not statistically significant and the results of multivariable regression analysis were not affected substantially after including husbands’ education and excluding participants without currently married. | This has been worked on and Table 3 has been revised to remove adjusted model 11 which model 11 has been attached as a supplementary file 2 introduced in the methods.  The abstract, results sections and discussion have been revised to focus mainly on model 1 as the main results. | Lines 36 – 47 and 205 – 206. |
| 3. (minor reporting issue) The authors stated that “Our results show an increase of 16 percentage points between the attendance of four or more ANC visits and health facility delivery.” in the Discussion section (Lines 256-). The revised manuscript has Supplementary Table 1, which reported the figures at the decimal point. The coverage of ANC four times or more was 59.9% and that of health facility delivery was 76.6%. The difference may be 16.7 percentage points. The sentence above may be “… an increase of 17 percentage points….” Please check it. | Thank you for pointing this out, we have revised it to 17% | 265 |
| **END** | | |
