# Supplementary material for: Continuum of care for maternal health in Uganda: A national cross-sectional study
Source: PLoS One. 2022 Feb 24;17(2):e0264190. doi: 10.1371/journal.pone.0264190 (PMC8870527; doi:10.1371/journal.pone.0264190)
Supplement: Attachment — Submitted filename: Respnse to reviewers comments -Revised.docx [file pone.0264190.s006.docx]

25^th^ /09/2021

To

Dr. Masamine Jimba

Academic Editor

PLOS ONE

Dear Dr. Masamine Jimba

Re; **Response to reviewers’ comments and resubmission of revised manuscript PONE-D-21-14873R2**

Thank you for taking off time to review and provide feedback on this manuscript titled **“Continuum of maternity care for maternal health in Uganda: a national cross-sectional study”**. Please receive the revised manuscript and a point-by-point response to the comments raised by the peer reviewers as summarized in the table below.

| **Comment** | **Response** | **Line** |
| --- | --- | --- |
| **Additional Editor Comments** | | |
| Thank you for your revision. I have checked your submission documents and found it very difficult to review it again. You are supposed to submit a set of clean and marked-up of the revised manuscript. However, 6 manuscripts are submitted and it is difficult to know which one to be reviewed. Please read the guideline and resubmit it again. | Sorry, about this difficulty. Next time I will delete all the previous versions from the system and only leave the revised ones. Otherwise, we appreciate your commitment to improve this manuscript. | NA |
| **Reviewer #1:** | | |
| Thank you for your continuous effort to revise this manuscript. I confirmed that the authors have addressed my previous comments. However, I would like to suggest some more revisions. | We thank you for the positive comments and appreciation of our efforts to improve this manuscript. | NA |
| According to the information on Revising Your Manuscript, a set of clean and marked-up copy of the revised manuscript should be submitted. However, a total of 6 manuscripts (3 were clean copies, and another 3 were marked-up copies) were compiled in the PDF for reviewer, which made me difficult to identify the one I should read this time. | We are sorry about this mix up. It will be better next time. | NA |
| Page 3. Please revise “22% seven million three hundred thousand (7.3 M)” to “22% (7.3 million)” | Thank for the suggestion, this has been revised | Line 96, page 3 |
| Page 4. Please delete a comma in the phrase of “A recent WHO report, estimates that” | This has been deleted | Line 115, page 4 |
| Page 5. Independent variables: I think current use of modern contraception should be removed from the regression model, because “current” use of contraception cannot predict the study outcome that happened in the “past”. Thus, the current use of modern contraception is not an appropriate independent variable in this model. (Sorry for pointing out at this time. I should have recognized and suggested earlier.) | Thank you for this very important observation and we agree with you. It has been removed from the regression model.  We have also revised table 3 and the rest of the manuscript | Line 158, page 5  Line 225 – 226 ,page 9 – 10 |
| Page 6. Please delete the following parts because it is already mentioned: “ (four or more ANC contacts, health facility utilization during childbirth and postnatal care attendance)” | Noted, this has been deleted | Line 187 – 188, page 6 |
| Page 6. Please report the percentage of complete continuum of maternity care for 2001, 2006, and 2011, respectively. | Thank you for the suggestion, but we chose not to report zeros for 2001 and 2006 UDHS, while for 2011 and 2016 it is implied and specifying it would be repetition. We think that referring readers to the supplementary table 1 is sufficient. | NA |
| Table 1, 2, and 3. Please use the same font type and size across the tables. | This has been corrected | Line 195 – 225  Pages 6-10 |
| Table1. Please delete 95%CI in the heading if actual data is not reported. | We have deleted it | Line 195, page 6 |
| Table 2. Please report all 95%CIs, or delete. | We have chosen to include all the 95% CI in this table | Lines 198 – 199, page 8 |
| Page 11. Regarding “Given the limitations associated with demographic health survey data, there is need for a longitudinal study to follow a cohort of women from pregnancy through childbirth to the end of the postnatal period,” I think a longitudinal study is not necessarily needed to investigate the reason behind the sharp drop of service utilization between childbirth and postnatal care. | Thank for this observation. Our suggestion of longitudinal cohort is in light of the fact that in the UDHS, participants interviewed may have used different components of the continuum of maternity care. For instance, a participant that never attended ANC and delivered at home may choose to attend postnatal care just to have her baby immunized. | Lines 262 -263, page 13 |
| **Reviewer #2:** | | |
| Dear authors, thank you for addressing the reviewer’s comments. The revised paper is much improved. Please find a few more minor comments below: | We thank you for the positive comments and appreciation of our efforts to improve this manuscript | NA |
| 1. Please report percentage only and delete the numerator and denominator indicated after the percentage in the first paragraph of the result section. For instance, please revise lines 219-221 “Among women who delivered at home, 29.4% (698/2382 95% CI: 28.0-31.7) had newborn PNC compared to 20.3% (1582/7780 95% CI: 19.2-21.0)” | We have deleted the extra information as suggested | Line 185 – 190, pages 5 – 6. |
| 2. Please review the first sentence in the conclusion: “In Uganda, less than one in ten (11%) women were able to utilise the entire continuum of maternity care for maternal and newborn health.” (lines 385-386). 11% is more than one in ten | Thank you for the observation, we agree. This has been revised to one in ten | Line 353, page 13 |
| **Reviewer #3** | | |
| Thank you very much for address the reviewer's comments made in the previous round. The authors addressed most of the comments. The point that the authors and the reviewer may not agree upon is the treatment of supply-side factors by a more detailed region variable. I understand that previous articles controlled for a geographic factor by a broad category of regional variable (East; North; West; and Central). Since the authors employed analyses that accounted for a complex survey design of DHS, the remaining issue is how detail the geographic unit (as a fixed-effect variable) would be in the analysis. While I think more detailed geographic variables could estimate the coefficients for other independent variables with narrower variance (and confidence interval) in addition to capturing more detailed geographic differences in CoC, I respect the authors' decision. | Thank you very much for your considered opinion. | NA |
| **END** | | |
