# Supplementary material for: Continuum of care for maternal health in Uganda: A national cross-sectional study
Source: PLoS One. 2022 Feb 24;17(2):e0264190. doi: 10.1371/journal.pone.0264190 (PMC8870527; doi:10.1371/journal.pone.0264190)
Supplement: Attachment — Submitted filename: Respnse to reviewers comments -December.docx [file pone.0264190.s007.docx]

21^st^ /12/2021

To

Dr. Masamine Jimba

Academic Editor

PLOS ONE

Dear Dr. Masamine Jimba

Re; **Response to reviewers’ comments and resubmission of revised manuscript PONE-D-21-14873R2**

Thank you for taking off time to review and provide feedback on this manuscript titled **“Continuum of maternity care for maternal health in Uganda: a national cross-sectional study”**. Please receive the revised manuscript and a point-by-point response to the comments raised by the peer reviewers as summarized in the table below.

| **Comment** | **Response** | **Line** |
| --- | --- | --- |
| **Additional Editor Comments** | | |
| After several revisions, the quality is getting better, but it still needs further revisions. Please respond to the following comments. | We appreciate all your efforts to improve this manuscript. | NA |
| The abstract of the cover page is an old one. Prevalence is still used, though it is corrected in the main text document. | We have cross checked this and the abstract is up to date. Prevalence appears only twice in the whole document and not in the abstract. | 14 – 43 |
| L50: I in 5,400… | This has been fully spelt out | 50 |
| L59: …maternity care… In L61, 62: …maternity healthcare… These two are the same or different in nuance? | We have chosen to stick to maternity care | 61- 62 |
| L75: …maternal child health… L78: maternal and child health (MCH)…These two are the same or different? | Noted, we have chosen to use maternal child health | 78 |
| L83: continuum of care for maternal health, L84: continuum of care, L86, 87: continuum of maternity care…The key words in this study is not appropriately used. | Sorry for this mix up, we have revised this to the study key words “continuum of maternity care” | 83 – 87 |
| L113: No need to abbreviate PNFPs. | This abbreviation has been deleted |  |
| L119: This sentence is based on a WHO report, but the reference is very old (9 years ago), and not the WHO report. | Thank you for the observation, it has been corrected | 469 |
| L123: ...secondary data analysis…L126, L131: …secondary analysis… | We have revised this to make it uniform “secondary data analysis” | 123 – 131 |
| L184…residents… | It has been corrected to “residents” | 184 |
| L188: The details are shown in… | Shown has been included | 189 – 190 |
| L189: 29.4%)? | This was not put in the table. In one of the previous reviews, one review asked for this and we added it in the text. PNC for home deliveries was 698/2372, 29.4 (28.0-31.7) while no PNC was 1674/2372 hence 70.6 (68.3-72.0). PNC for health facility delivery was 1582/7780, 20.3 (19.2-21.0) and NO PNC was 6199/7780, 79.7 (79.0-80.8) | NA |
| L189: …table 1 L190: …Table 2 table of Table? | This is not clear to this, but it is probably related to the table above | 209 – 210 |
| L197, L221: Tables 2, 3. The use of capital letters are not appropriate. | This has been revised and capital letters removed from table 2 and 3. | 233 – 235 |
| L224: Discussion is still redundant and confusing. The contents are getting better, but it is not easy to follow your discussion as paragraph wiring is very weak. Please try to include only one topic for each paragraph and do not discuss many different issues in each paragraph. Please make a summary of your main findings in the first paragraph of discussion section and do not discuss them. Then from the second paragraph, show your unique finding first in the first sentence of each paragraph as much as possible, and discuss your finding using your data and references. The flow should be specific to general. | We have revised this section accordingly to improve the flow. | 224 – 380 |
| L292.293, 299: Eastern region… Western region? Central region? | The capital letters have been revised | 292 |
| L343: Both maternal and newborn health? Previously, newborn health was not included. Why it suddenly appears here? | Sorry, “newborn” has been removed as requested earlier. | 343 |
| L373: Reference is still not well done. For example, for ref 3, Lancet Glob Heal. Please check this section again. | We have revised this section | 374 – 572 |
| **Journal requirements** | | |
| Please review your reference list to ensure that it is complete and correct. If you have cited papers that have been retracted, please include the rationale for doing so in the manuscript text, or remove these references and replace them with relevant current references. Any changes to the reference list should be mentioned in the rebuttal letter that accompanies your revised manuscript. If you need to cite a retracted article, indicate the article’s retracted status in the References list and also include a citation and full reference for the retraction notice. | Thank you, we have revised and updated our reference list | 374 – 572 |
| **Reviewer #1:** | | |
| Thank you for addressing my previous comments. I saw the revised manuscript is much improved. Please find a few more minor comments. | We appreciate all your efforts to improve this manuscript. | NA |
| Table1. I am wondering if the authors might have forgot to delete the descriptive statistic of contraceptive use. | Sorry about this oversight, we have deleted it | 194 – 197 |
| Table2. Please add a heading for frequency, percentage, and 95% confidence interval so that the term of 95% confidence interval in each cell can be deleted. | We have added this information | 197 – 198 |
| Tabl3. Please add a heading for frequency and percentage or (n, %) at the top of the columns of Incomplete CoC and Complete COC. | This has been added | 222 – 223 |
| L274 in the clean file: Please delete current use of modern contraceptives. | Sorry about this oversight, we have deleted it | 274 |
| **Reviewer #2:** | | |
| I thank the authors for addressing my comments. They addressed all the comments, and the manuscript improved. | We appreciate all your efforts to improve this manuscript. | NA |
| **Reviewer #3** | | |
| Overall comment; Overall, the authors discussed the important topic of the continuum of care for maternal and newborn health in Uganda. They conducted a secondary analysis of the 2016 Demographic and Health Survey to determine the prevalence and factors associated with the continuum of maternity care in Uganda. There was a general lack of interpretation of results regarding the discontinuities between antenatal care, health facility childbirth, and postnatal care services. The associated factors, such as initiation of ANC in the first trimester, level of education, and exposure to mass media, have been reported in several studies. Therefore, it would be great if the authors could add evidence and implication for policy makers based on findings of this study. Please find my comments in blue. | -We appreciate all your efforts to improve this manuscript.  -Thank you for the observation regarding the discontinuities in care. We were concerned about the same finding, so we tried to explain and interpret the same in the discussion. | 253 – 289 |
| Abstract |  |  |
| Results: About 59.9% (6080/7262) (95% CI: 59.0-60.8) had four or more antenatal visits. -Please check if the denominator is correct. I think you do not need to report exact numbers every time if you report results with a percentage with the denominator. | Noted the denominator should be 10,152.  We have chosen to delete the denominator, since it is uniform across the results reported. | 28 – 31 |
| Introduction | | |
| The first paragraph seems too long. It needs to be restructured to provide an overview of maternal, neonatal and under-5 childhood deaths by comparing sub-Saharan Africa with the other regions of the world. | We have revised this section as advised | 45 – 59 |
| Almost all 277,300 (94%) of the annual maternal deaths occur in low and middle-income countries and roughly two-thirds (196,000) of these deaths happen in South East Asia and sub- Saharan Africa [2,3]. - How many annual maternal deaths occurred? When you report percentages, please indicate their denominators together. | Thank you for this observation, we have revised it accordingly. | 45 – 49 |
| It is estimated that 45% of all the under-5 childhood deaths happen early in the neonatal period, especially in the first seven (7) days of life. - Is it the global situation or and sub-Saharan Africa? | These are global deaths | 55 |
| In introduction, you need to describe the situations of Uganda in terms of maternal, neonatal and under-5 childhood deaths if available. It is also recommended to include country data related to the study objectives briefly, such as the number of women of reproductive age, ANC and PNC attendance. It would provide rationales for the study objectives and the interventions for the continuum of maternity care recommended in the discussion. | Thank you for the advice, we have added this information to the introduction.  -Country data related to study objectives was provided under the section on study setting | 54 – 57  96 – 125 |
| The current study focused on the completion of the continuum of care by specifically looking at antenatal care (ANC) attendance, health facility utilization during childbirth, and postnatal care (PNC). – This sentence in the second paragraph should be moved to the next paragraph as it describes the study objectives. | We feel that this sentence is in the right place as per your suggestion. | 88 – 90 |
| It is better to include evidence available on the association between the continuum of maternity care and maternal and newborn health if available in the second paragraph. | Thank you, this is provided in the introduction | 61 – 62  84 – 87 |
| Materials and Methods | | |
| Study design: it should be mentioned that this study is the secondary analysis of the nationally-representative cross-sectional study (UDHS) conducted between June and December 2016. | We have indicated this, thank you | 123 – 126 |
| Sample size and sampling: Did all the sample of 10,152 women have the outcome data? If you excluded some women due to missing data on the outcome, please explain it or add a study population flow chart. | We only had one variable that had missing data fit for multivariable analysis and how missing data was handled was explained in the statistical analysis.  Furthermore, the missing data was also indicated at the end of table 1 | 178 – 182 |
| Outcome Variable: Complete continuum of care was the outcome variable. Complete continuum of maternity care was considered when a woman reported having had: at least four ANC (ANC4+) contacts during the most recent pregnancy, utilized a health facility during the most recent childbirth and had at least one postnatal check-up within six weeks after childbirth: It would be great if you could add definitions of each segment consisting continuum of care. Are they in line with the Uganda MOH/WHO’s recommendations, for instance, in terms of the number and quality of ANC visits, supervision of a skilled service provider and length of stay in a health facility during childbirth and timing of postnatal check-up? | Thank you for the comment and observation. We aimed at utilization of the available services, and not the quality of care, so we did not analyze content/quality/timing of ANC, PNC. This information is provided under the outcome variables section. | 140 -149 |
| Independent variables: Some variables included in the analyses and the result tables were not listed here, such as contraceptive use and religion. For variable selection, it is better to refer to a conceptual framework, such as Owilli’s continuum of care framework for maternal, newborn and child health (https://doi.org/10.1186/s12889-016-3075-0). | -We included all the variables in the methods section as they are in table1 and 3.  - Religion in the methods section has been highlighted. Regarding contraceptive use, we were advised to drop this from the study by one of the reviewers in the last review.  - Use of Owilli’s continuum of care framework was advised in the earlier reviews and its indicated in the statistical analysis. | 162 – 185 |
| Results | | |
| Characteristics of the study population: If you describe 95% CI in the narrative part, it is better to include them in Table 1 as well. Also, recheck Table 1 as total numbers exceed the sample size for some variables (i.e., postnatal care, residence). | -We have cross checked our table 1, and all variables including residence and postnatal care in table 2 add up to 10,152.  -We don’t think that it is necessary to include confidence intervals in table 1 because the table will become congested and it is not standard practice | 198 – 200 |
| Of the 10,125 women, 59.9% (6,080/7262) (95% CI: 59.0-60.8) had four or more ANC contacts while 76.6% (7,780/10,125) (95% CI: 75.8-77.5) utilized health facility for childbirth and 22.5% (2,280/10,125) (95% CI: 21.5-23.2) attended postnatal care within six weeks after childbirth. – Is the denominator for four or more ANC visits correct? | YES, it is, the denominator for all these percentages is 10,125. What is shown in the brackets are the numerators. | 183 – 192 |
| Factors associated with complete continuum of maternity care: The description is too short. You need to explain the key statistical outputs provided in Table 2. Which model is the main model that you used to discuss the results? In the description, were those independent variables associated with the outcome negatively or positively? For this, I think it is important to include odds ratios for some key independent variables. | Table 2 results were discussed under characteristics of the study population. This section of results focused on statistical outputs of table 3 and we included the main model in the statistical analysis section and also in the first statement of this section. We ensured to use ***more/less likely to have complete continuum of maternity care*** to show the positive/negative association. We included the odds ratios of all the significant variables under this section. | 186 – 199  202 – 219 |
| Throughout the manuscript, there were inconsistencies in reporting formats and typos, such as the use of thousand separators. Please review the whole manuscript. | We have taken time to revise correct all these typos and other related errors. Quraish, please correct what you can see as well. | NA |
| Discussion | | |
| We found a low level of utilization of complete continuum of maternity care at 11%. The level of use was highest for the place of childbirth at (7,789/10,125):77%), followed by ANC 4 or more visits at (6,080/7,262: 60%) and lowest for PNC at (2,280/1, 0125: 23%). - The discussion of the study seems mixed with results. This sentence was already presented in the results. You need to add more interpretation of the findings in the discussion. | Thank you for the advice, we have done this in the revised manuscript | 242 – 368 |
| Uganda’s levels of completion of the continuum of maternity care is similar to that of other countries in the region. For both Ethiopia and Tanzania, the reported level is at about 10% [27,29], 8% for Ghana [28] and 7% for Lao [12]. Compared to Kenya, it is more than three times below the reported level of completion of the continuum of maternity care at 36% [32]. - You compared the prevalence of a complete continuum of maternity care among several countries. Did these studies use the same definition of the continuum of maternity as one used for this study? Moreover, are they all national-level studies? Please note the level of continuum of care may vary even within a country. Therefore, if you want to compare the prevalence of continued care with other studies, they should be nationally representative data. Otherwise, please specify locations, for instance, districts and regions. | Thank so much for this important observation.  Yes, we paid attention to these differences in the discussion as we made comparisons with other countries. | 230 – 246 |
| The largest dropout of 54% occurred between the stages of childbirth and the postnatal period. - I think it would be useful if you could provide more insights on this point by comparing it with the situations of other counties. For instance, how about the situation in Kenya? | Note, we have done as advised in the revised manuscript. | 248 – 254 |
| In the health sector, the decentralization policy has greatly improved physical access because it is estimated that 72% of the population lives within five kilometers of a health facility [34].This could partly explain the observed increase in the number health facility deliveries because the basic emergency obstetric care services have been brought closer to the community. - This paragraph is confusing. This study used only UDHS 2016 data, so you were not able to explore changes over time. If you want to discuss the increase in the number of health facility deliveries, you need to present the baseline. You did not compare longitudinal data and present any data about health facility delivery in the past in this manuscript. Decentralization policy may have increased all the ANC, health facility deliveries, PNC. This policy was not discussed before, so you need to explain this. | Thank you for this observation, it is correct.  However, in response to comments from a reviewer in the previous round we provided a supplementary file showing trends in the level of continuum of care over the years. We refer to it in the first paragraph of this section. | 232 – 232 |
| Our results show an increase of 16% between the attendance of four or more ANC visits and health facility delivery… On the other hand, the observed drop in utilization between childbirth and postnatal care could be a pointer to the quality of service received during childbirth and labour. - They are directly related to the continued care. It would be useful if you could elaborate on why these discontinuities occurred, apart from the poor quality of maternity care in Uganda. | Thank you, we have suggested possible explanations for these discontinuities | 248 – 284 |
| Most of the factors associated with the continued care, such as initiation of ANC in the first trimester, level of education, and exposure to mass media, were reported and examined in previous studies. It is great if you could add evidence to them based on the findings of this study. At the same time, you described each associated factor using one paragraph, which is a bit wordy. It is great you can summarize discussions and put more focus on the unique findings of your study. | -Thank you so much for this comment. As much as other countries have looked at factors associated with continuum of care, we believe we cannot assume that factors shown in other studies apply to the Ugandan context. We have not come across any study in Uganda that has analyzed DHS data to assess factors associated with continuum of care.  -Furthermore, we have discussed the dropout between ANC and facility delivery and facility delivery and PNC.  -The key finding in this study is about the discontinuities at the different stages of care, which we have discussed extensively and identified gaps that we could not answer using the UDHS data. | 229 – 373 |
| You found large geographic variations in the continuum of maternity care. It is interesting if you can further investigate why the Eastern area, the poorest region with some of the poorest maternal and child health indicators, had a better continuum of care than the Western area. For instance, are there development partners supporting the continuum of care in this region? | This tends to attract more development partners in the region to support the provision of social services such as maternity care. | 327 – 328 |
| This finding may partly be explained by the unique cultural practices and beliefs about pregnancy and childbirth in this region – Please provide a few examples here. | This is one the examples that we have provided; “For instance, anecdotal evidence from this region suggests that for the first seven days after childbirth, the baby should not be seen by strangers. So, babies born at home are kept indoors until the first week of life has elapsed. While those that give birth normally in health facility are in a rush to get back home early to full fill this cultural practice because the health facility does not offer adequate privacy.” | 321 – 328 |
| I think it would be great if you could include recommendations beyond mass media and education programs to promote the complete continuum of care, such as support for early initiation of ANC and the integration of ANC and PNC programs based on your findings. | These have been included in the abstract and conclusion sections | 41 – 43  421 – 423 |
| **END** | | |
