# Supplementary material for: Continuum of care for maternal health in Uganda: A national cross-sectional study
Source: PLoS One. 2022 Feb 24;17(2):e0264190. doi: 10.1371/journal.pone.0264190 (PMC8870527; doi:10.1371/journal.pone.0264190)
Supplement: Attachment — Submitted filename: Response to reviewers comments V5.docx [file pone.0264190.s009.docx]

27^th^ /01/2022

To

Dr. Masamine Jimba

Academic Editor

PLOS ONE

Dear Dr. Masamine Jimba

Re; **Response to reviewers’ comments and resubmission of revised manuscript PONE-D-21-14873R5**

Thank you for taking off time to review and provide feedback on this manuscript titled **“Continuum of maternity care for maternal health in Uganda: a national cross-sectional study”**. Please receive the revised manuscript and a point-by-point response to the comments raised by the peer reviewers as summarized in the table below.

| **Comment** | **Response** | **Line** |
| --- | --- | --- |
| **Additional Editor Comments** | | |
| Thank you for your revision for this time. The quality of manuscript is much better than before. Please make more efforts to improve it much better. Your basic writing skills have improved, but there is a room for further improvement. | Thank you for the encouragement and for your time and effort to make this manuscript better. We do not take it for granted. | NA |
| 1. L25: …multivariable logistic regression analysis to determine… | We have added the missing word ‘analysis’ | 25 |
| 2. L37: Eastern Uganda, and exposure to … | We have added the missing word ‘and’ | 37 |
| 3. L48: Ref [4]. Please check if this reference really correct. The reference title suggests that this is not a correct citation. Please check all the references, too, if they are correctly cited. | This has been revised | 48  388 – 616 |
| 4. L67: ...at least eight antenatal care (ANC)…Please abbreviate it when it first appears. This is a basic rule for using abbreviation. | Thank you for this observation, in L24 that is when ANC first appears in this manuscript and believe that it is correctly done. | 68 |
| 5. L79: …unsatisfactory maternal and child health (MCH)…. | We have added the missing word ‘and’ | 79 |
| 6. L80-81: …such as ANC, skilled…. | We have corrected this | 81 – 82 |
| 7. L84: ….looking at ANC…Once it is abbreviated, no need to do it again. | We have corrected this | 85 |
| 8. L85: …childbirth and PNC. We aimed…. | We have corrected this | 86 |
| 9. L92: The Uganda Demographic and Health Survey (UDHS)… | We have corrected this | 93 |
| 10. L123: …the secondary data analysis of… | We have added the missing word ‘data’ | 124 |
| 11. L124 or L125?: Study population… Please check the text carefully. | Sorry, this should be a separate subtitle. We have corrected it. | 126 |
| 12. L138-139: …maternity care. Its definition was based on the WHO…. | Thank you for the suggestion, we have revised it accordingly. | 140 – 141 |
| 13. L145: What is CoC? | We have revised it now. | 147 – 148 |
| 14. L169: …conducted a bivariate logistic regression analysis…Please add “analysis” for other similar parts. | We have added the missing word ‘analysis’ | 25, 172, 203 |
| 15. L177:…(AOR), 95% CI and p-values… | Corrected | 180 |
| 16. L187: …(2,280)… Please close a parenthesis appropriately. | We have closed it appropriately | 190 |
| 17. L188: …(1,091) … Please close a parenthesis appropriately. | We have closed it appropriately | 191 |
| 18. L200:…regression analysis… | We have added the missing word ‘analysis’ | 203 |
| 19. L229-230: This sentence suggest that you have data about continuum of maternity care in 2011. So, this study is not the first time showing the level of the completion of the continuum of maternity care? Please clarify this point. | We have presented that data for 2011 in the supplementary file. | 234 |
| 20. L229: Please show that the completion rate was about 11%. | We have revised this sentence to reflect this | 232- 233 |
| 21. L239: This could be partly because…This sentence does not show Ghana and Lao PDR are similar. They are also middle income countries. | We have checked this again and these two countries are not yet middle-income countries.  <https://opendevelopmentmekong.net/news/world-bank-classifies-laos-as-lower-middle-income-economy-for-2020/>  <https://mofep.gov.gh/press-release/2021-04-14/ghana-has-not-been-down-graded-as-a-low-income-country> | NA |
| 22. L240: (GDP), while… | We have closed the parenthesis appropriately. | 245 |
| 23. L242: World Bank…Please be more careful about the use of capital letters. | Sorry about this, it has been corrected | 246 |
| 24. L245, 246: Is this sentence your finding? This paragraph is too long. Please show your finding first and do not over discuss it. You may cut many sentences in this paragraph. | Yes, this is our finding. We have revised this paragraph as suggested by cutting out several sentences. | 249 - 293 |
| 25. L302, L313: “central region” or “Central Uganda”? | Sorry about this, it has been corrected | 315 |
| 26. L303: “eastern region” or “Eastern Uganda”? | Sorry about this, it has been corrected | 316 |
| 27. L303, L310:“western Uganda” or “Western Uganda”? | Sorry about this, it has been corrected | 316 |
| 28. L305: …region of the country. Therefore… | Sorry about this, it has been corrected | 318 |
| 29. L312: …the country and its MCH indicators… | Corrected appropriately | 326 |
| 30. L320: …the poorest MCH indicators… | Corrected | 334 |
| 31. L341: …for better MCH in the country. Sometimes MCH covers newborn health in some context. | Thank you for the clarification, we have corrected this | 354 |
| 32. L366: In Uganda, about 11% of women completed the continuum of maternity care,… | Thank you for the suggestion, we have incorporated it | 379 |
| 33. L368:…utilization of PNC services. | We have corrected this. | 381 |
| 34. L373:…integration of all MCH services may go a long way in improving the continuum of maternity care. | We have corrected this. | 387 |
| 35. L375-6:…towards improved MCH. | We have corrected this. | 389 |
| 36. L412: Check the journal name again. | We have corrected the name of the journal, thank you for the observation | 425 |
| 37. L415: Udhs or UDHS? | This has been corrected now | 427 |
| 38. L497: Check the abbreviation | We have checked it | 510 |
| 39. L501. Check the abbreviation. | We have checked it | 514 |
| 40. L508: Check the abbreviation. | We have checked it | 521 |
| 41. L544: Check the abbreviation. | We have checked it | 557 |
| **Reviewer #1:** | | |
| Thank you for addressing my previous comments. I have one last comment on Table 2. | We are very grateful for all the time and effort that you have dedicated to make this manuscript better. | NA |
| Let me ask once again for deleting "95%CI" before 59.0, 39.2, 75.8, 22.5, 21.5, 76.8, 10.0, and 88.8.  Because you have indicated (95%CI) at the heading of the table, readers understand that (95.0-60.8) indicates 95%CI. | Okay, sorry about this inaction. We totally agree with your suggestion. I think the track changes were too many in the previous version and we could not easily see this repetition | 200 – 201 |
| Please look at Table 3 in which (95%CI) is indicated at the heading of the table only, not for each result. This is what I requested for Table 2.  In this way, please keep in your mind that consistency across a manuscript is important for us researchers to deliver our findings appropriately. | Point taken, thank you very much for consistently pointing this out. |  |
| **END** | | |
