# Supplementary material for: Continuum of care for maternal health in Uganda: A national cross-sectional study
Source: PLoS One. 2022 Feb 24;17(2):e0264190. doi: 10.1371/journal.pone.0264190 (PMC8870527; doi:10.1371/journal.pone.0264190)
Supplement: Attachment — Submitted filename: Response to reviewers comments.docx [file pone.0264190.s010.docx]

07^th^ /02/2022

To

Dr. Masamine Jimba

Academic Editor

PLOS ONE

Dear Dr. Masamine Jimba

Re; **Response to reviewers’ comments and resubmission of revised manuscript PONE-D-21-14873R6**

Thank you for taking off time to review and provide feedback on this manuscript titled **“Continuum of maternity care for maternal health in Uganda: a national cross-sectional study”**. Please receive the revised manuscript and a point-by-point response to the comments raised by the peer reviewers as summarized in the table below.

| **Comment** | **Response** | **Line** |
| --- | --- | --- |
| **Additional Editor Comments** | | |
| Thank you for your revision. Though most of comments were well-addressed, it still needs further revisions: one major and several minor ones. | Thank you for the encouragement and for your time and effort to make this manuscript better. We do not take it for granted. | NA |
| L240: Please read the World Bank home page (or a blog) carefully.  <https://blogs.worldbank.org/opendata/new-world-bank-country-classifications-income-level-2020-2021>  “The World Bank assigns the world’s economies to four income groups—low, lower-middle, upper-middle, and high-income countries.” Middle income countries are divided into two：lower middle and upper-middle. So lower-middle income countries are aprt of middle income countries and it is different from low income counties.  You quoted two references in your response sheet. a. “The World Bank has classified Laos as a lower-middle-income economy in its latest classification for the 2020 fiscal year.”  <https://opendevelopmentmekong.net/news/worldbank-> classifies-laos-as-lower-middle-incomeeconomy- for-2020/  b. Ghana has not been down-graded as a low income country. <https://mofep.gov.gh/press-release/2021-04-> 14/ghana-has-not-been-down-graded-as-a-lowincome- country. So both are middle income countries. | Thank you for the clarification, this paragraph has been revised and we think that the anomaly has been corrected. | 235 – 256 |
| L356: …completed the continuum of maternity care. In academic literature, an expression “maternal care” is often used. Please check which is better “maternity care” or “maternal care,” throughout the manuscript. | We have revised this throughout the document | NA |
| L386: Lancet Glob Heal should be Lancet Glob Health. | Revised | 399 |
| L400: As other references are abbreviated, this journal name should be “correctly” abbreviated. | Revised | 413 |
| L485: World Heal Organ. It should be World Health Organization. | Revised | 498 |
| L496: BMJ Glob Heal should be BMJ Glob Health. | Revised | 509 |
| L552: BMJ Glob Heal should be BMJ Glob Health. | Revised | 565 |
| L577: Front Public Heal should be Front Public Health. | Revised | 590 |
| L586: Communicatio. Is it correct? | Revised | 599 |
| L604: Trop Med Int Heal should be Trop Med Int Health. | Corrected | 608 |
| Please check references one by one with your own eyes without depending on computer soft wares. | Thank you for the advise this has been done | NA |
| **END** | | |
